# Simple Repeat-Primed PCR Analysis of the *Myotonic Dystrophy Type 1* Gene in a Clinical Diagnostics Environment

**DOI:** 10.1155/2013/857564

**Published:** 2013-11-11

**Authors:** Philippa A. Dryland, Elaine Doherty, Jennifer M. Love, Donald R. Love

**Affiliations:** ^1^Diagnostic Genetics, LabPlus, Auckland City Hospital, P.O. Box 110031, Auckland 1148, New Zealand; ^2^School of Biological Sciences, University of Auckland, Private Bag 92019, Auckland 1142, New Zealand

## Abstract

Myotonic dystrophy type 1 is an autosomal dominant neuromuscular disorder that is caused by the expansion of a CTG trinucleotide repeat in the *DMPK* gene. The confirmation of a clinical diagnosis of DM-1 usually involves PCR amplification of the CTG repeat-containing region and subsequent sizing of the amplification products in order to deduce the number of CTG repeats. In the case of repeat hyperexpansions, Southern blotting is also used; however, the latter has largely been superseded by triplet repeat-primed PCR (TP-PCR), which does not yield a CTG repeat number but nevertheless provides a means of stratifying patients regarding their disease severity. We report here a combination of forward and reverse TP-PCR primers that allows for the simple and effective scoring of both the size of smaller alleles and the presence or absence of expanded repeat sequences. In addition, the CTG repeat-containing TP-PCR forward primer can target both the *DM-1* and *Huntington disease* genes, thereby streamlining the work flow for confirmation of clinical diagnoses in a diagnostic laboratory.

## 1. Introduction

Myotonic dystrophy type 1 (DM-1) is a multisystem disorder with a highly variable phenotypic expression. This autosomal dominant neuromuscular disorder has been reported to cause myotonia, progressive muscle weakness, and atrophy in skeletal muscles, as well as cardiomyopathy, cataracts, and dysfunction in the endocrine and central nervous systems [1, 2].

DM-1 is caused by a CTG trinucleotide repeat expansion in the 3′ untranslated region of the *myotonic dystrophy protein kinase* (*DMPK*) gene, which is located on chromosome 19q13.3. The number of repeats a patient carries is associated with clinical severity. Repeats of 5–34 are found in unaffected individuals, and those that lie in the range of 35–49 are considered abnormal premutations such that carriers are at risk of having affected offspring with larger repeat lengths. Patients with DM-1 are typically classified into three overlapping groupings based on the number of repeats, the severity of clinical presentation, and the age of onset. These groupings are mild, classical, and congenital with repeat sizes ranging from 50 to 150 repeats, 100 to 1000 repeats, and >2000 repeats, respectively [3, 4]. Despite these repeat ranges, the stratifying of patients into classical and congenital categories can pose difficulties as some congenital patients carry CTG repeats that overlap with the classic range. In addition, the situation is further confounded by the recent identification of a juvenile category with CTG repeats that lie at the high end of the classic range but with a different clinical severity [5, 6].

The confirmation of a clinical diagnosis of DM-1 involves the molecular genetic testing of the *DMPK* gene. This genetic testing is commonly performed by PCR amplification of the CTG repeat-containing region of the *DMPK* gene, and subsequent determination of amplicon lengths using capillary-based electrophoresis. This method is only able to identify patients that fall within the mild range, so any sample showing apparent homozygosity has usually involved a second round of testing by Southern blot (SB) analysis to detect the presence or absence of larger repeat lengths [3, 7].

**Figure 1:**
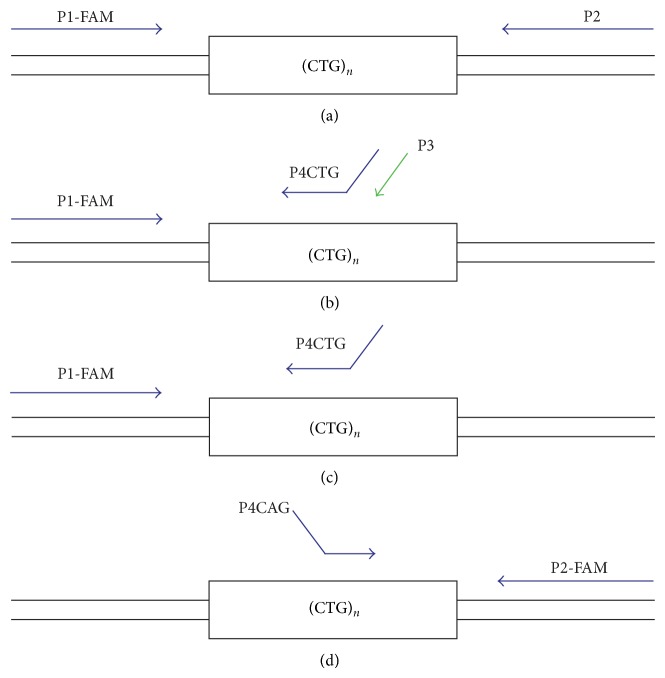
Location of primers for each of four amplifications of the CTG repeat region of the *DMPK* gene.

Warner et al. (1996) suggested the use of a triplet repeat-primed PCR (TP-PCR) to replace Southern blotting as a means of detecting hyperexpansions of trinucleotide repeat sequences [8]. TP-PCR reduces the potential turnaround time for a test from a week to one day, as well as the cost of testing [8, 9]. TP-PCR usually uses three primers (Figure 1): one that is fluoresceinated and flanks the repeat region (P1-FAM), a second that is complementary to the targeted repeat but carries a nonspecific tail sequence (P4), and a third that is identical to the nonspecific tail sequence (P3). The P4CTG primer is added at 1/10th the concentration of P1-FAM and P3 primers, while the P3 primer is designed to prevent progressive shortening of the amplicons during PCR cycling [8]. TP-PCR produces a characteristic profile of amplicons of increasing length, which differ by the length of a repeat unit (3 bp), but of diminishing yield [10]. This profile enables the rapid identification of large pathogenic CTG repeats that cannot be amplified using primers that flank the repeat region. The main disadvantage of TP-PCR is that it does not enable the repeat number to be determined [11]. This disadvantage has no serious clinical implications in the case of myotonic dystrophy as the age of presentation plays a pivotal role in helping a diagnostic laboratory interpret the implications of CTG repeats in excess of 150.

Critically, recent evidence has shown that TP-PCR can lead to false negative results in 3%–5% of DM-1 cases. This outcome is due to sequence interruptions (comprising CCG, CTG, and GGC sequences) that lie within the 3′ end of an expanded CTG repeat tract [2, 8, 9, 12], which appear to prevent binding of the repeat (P4) primer. In order to address this problem, Radvansky et al. (2011) has described a bidirectionally labeled TP-PCR method in which amplification products are anchored at the 3′ end of a CTG repeat expansion rather than the 5′ end [11]. The effect of this redesign is that it overcomes the failure in detecting expansion-positive patients carrying repeat interruptions.

Despite the above developments, we sought to simplify TP-PCR further by relying on only two rather than three primers, refining the amplification conditions to be more aligned with those used routinely in a diagnostic laboratory, and having the flexibility to allow hyper-expansions to be detected in other triplet repeat disorders.

## 2. Materials and Methods

Twenty-one genomic DNAs that had been analysed by conventional PCR and Southern blotting were used to validate the proposed TP-PCR methodology. These DNAs comprised seven unaffected control individuals with repeat sizes ranging from 4 to 27 and 14 samples carrying expanded CTG repeats ranging from 54 to over 1000. Fifth-three further DNAs were analysed in a diagnostic setting by conventional PCR and the validated TP-PCR methodology. These DNAs comprised of 25 unaffected controls with repeat sizes ranging from 4 to 27 and 28 samples carrying expanded CTG repeats ranging from 50 to full expansions.

The initial 21 DNA samples were tested using four variations of PCR amplification, as shown in Figure 1. The first comprised conventional PCR using P1-FAM forward and P2 reverse primers. The second was a TP-PCR method using the P1-FAM forward primer with the P4CTG reverse primer at 1/10 the concentration and the P3 reverse primer specific to the tail region of primer P4CTG (TP-PCR forward primer combination, together with primer P3). The third was the same as the second but only used P1-FAM forward and the P4CTG reverse primers at the same concentration (TP-PCR forward primer combination). The final PCR method used the P2-FAM reverse primer with a P4CAG forward primer (TP-PCR reverse primer combination). Primer sequences are shown in Table 1. The third and fourth PCR methods (TP-PCR forward and TP-PCR reverse primer combinations) were validated (in triplicate) and used in conjunction with conventional PCR to analyse 53 further DNA samples.

Each 25 *μ*L PCR comprised FastStart Reaction Buffer without MgCl_2_ (Roche), 2 mM MgCl_2_ (Roche), GC-rich solution (Roche), 10 mM dNTP mix, 20 *μ*M forward and reverse primers, 1U FastStart Taq DNA Polymerase (Roche), and 50 ng of genomic DNA. The PCR amplification conditions comprised an initial denaturation of 94°C for five minutes then 35 cycles of denaturation at 94°C for 45 seconds, annealing at 70°C for 30 seconds with extension at 72°C for 30 seconds and a final extension at 72°C for 10 minutes. The conventional PCR used a longer extension time of 2 minutes in order to reduce the presence of split peaks, which were not observed in TP-PCR profiles. PCR products were subjected to capillary electrophoresis using an Applied Biosystems model 3130xl Genetic Analyzer, and the data were analysed using GeneMapper software.

## 3. Results

An initial validation study was undertaken using 21 genomic DNA samples. Amplification of the CTG repeat region of the *DMPK* gene involved four different reactions. The first used primers P1-FAM and P2, which flank the repeat. The second, designated the TP-PCR forward primer combination, comprised P1-FAM and P4CTG. The latter primer, also termed HD3, had originally been designed as the reverse primer for amplification of the CAG repeat region of the *Huntington disease* (*HD*) gene [6] and comprised a 3′ (CTG)_4_ repeat and a 5′ tail of a 17-base sequence immediately downstream of the *HD* gene repeat. Amplification using the TP-PCR forward primer combination was undertaken in the presence and absence of primer P3. The sequence of this primer corresponded to the 17-base 5′ tail of primer P4CTG. Finally, the TP-PCR reverse primer combination was used that comprised primers P2-FAM and P4CAG; the latter primer was similar in sequence to primer P4CTG except that the (CTG)_4_ repeat was replaced by a (CAG)_5_ repeat.

**Figure 2:**
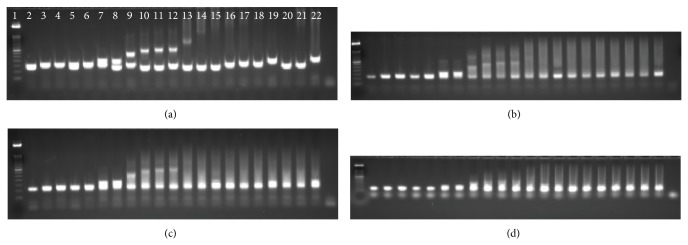
Agarose gel electrophoresis of PCR products for each of four amplifications of the CTG repeat region of the *DMPK* gene. (a) Primers P1-FAM and P2; (b) primers P1-FAM and P4CTG (TP-PCR forward primer combination) with P3 primer; (c) TP-PCR forward primer combination; (d) P2-FAM and P4CAG (TP-PCR reverse primer combination). From left to right the gels show DNAs with repeat lengths of [7,11], [11,13], [12,13], [5,14], [11,15], [11,26], [5,27], [13,54], [5,74], [4,77], [11,84], [5,120], [5,250], [5,380], [12,500], [14,530], [12,700], [1000–1500], [1067–1600], [5,405], and [24,260–490].

**Figure 3:**
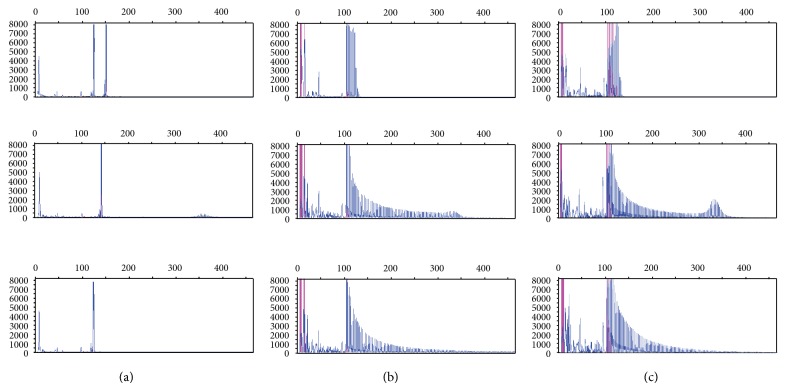
Electropherograms of PCR products for each of four amplifications of the CTG repeat region of the *DMPK* gene. (a) Primers P1-FAM and P2; ((b), (c)) TP-PCR forward primer combination with and without the P3 primer, respectively. From top to bottom the electropherograms are from DNAs with repeat lengths of [5, 14], [11, 84], and [5, 1600].

The amplification products of all PCRs were initially analysed by gel electrophoresis (Figure 2). Conventional PCR using primers flanking the CTG repeat region amplified up to 150 CTG repeats, which would be appropriate to confirm a clinical diagnosis of mild DM-1 (50–150 repeats). The TP-PCR primer combinations (forward and reverse), with and without the additional P3 primer, amplified both CTG alleles in all patients (except those carrying expanded alleles in excess of 150 CTG repeats), but against high background staining. Critically, the presence of the P3 primer resulted in qualitatively weaker amplification of CTG repeat alleles compared to the absence of this primer (Figure 3).

**Figure 4:**
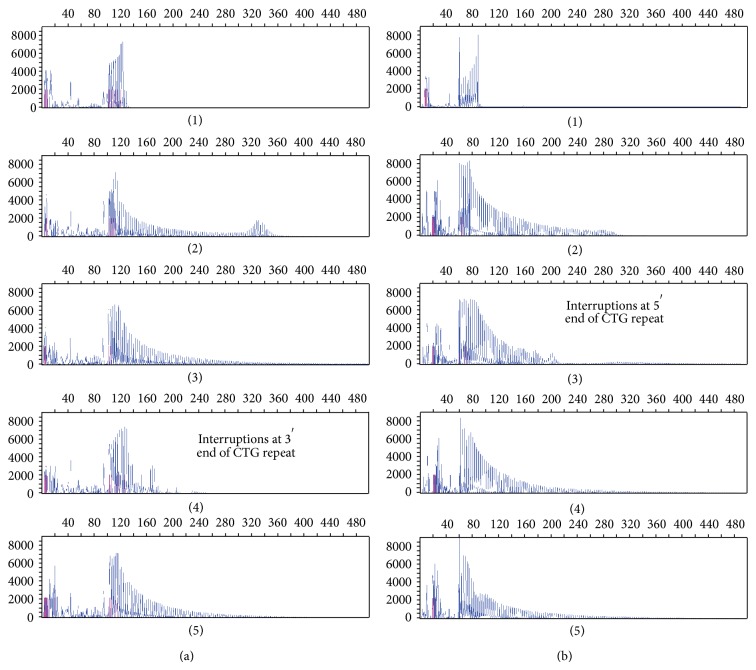
Electropherograms of PCR products using the TP-PCR forward and reverse primer combinations. (a) and (b) show electropherograms derived from using the TP-PCR forward and reverse primer combinations, respectively. From top to bottom the electropherograms are from DNAs with repeat lengths of [5, 14], [11, 84], [5, 380], [5, 250], and [5, 1600].

The TP-PCR forward primer combination, with and without P3, was unable to accurately amplify the characteristic ladder profile for two patients with expanded repeat sequences resulting in false negative results; an example of this is shown in Figure 4(a)(4). Amplification using the TP-PCR reverse primer combination, without primer P3, was able to detect the presence of the expanded repeat sequence (Figure 4(b)(4)). Profiles of 3′-interrupted sequences are expected to show a characteristic ladder profile, with peaks increasing in length by 3 bp increments but diminishing in intensity with length. A break in this profile would be expected if the repeat was interrupted, after which the ladder should be reinstated with diminishing yield associated with increasing CTG repeat length.

The converse to the above was also found in which a TP-PCR forward primer combination gave a characteristic hyper-expansion profile (Figure 4(a)(3)), but the TP-PCR reverse primer combination suggested interruptions at the 5′ end of the CTG repeat (Figure 4(b)(3)), hence supporting the need to use both the TP-PCR forward and reverse primer combinations to detect repeat hyper-expansions.

**Figure 5:**
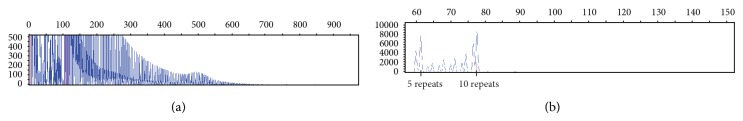
Electropherograms of PCR products using the TP-PCR forward and reverse primer combinations illustrating sizing capabilities. (a) and (b) show electropherograms derived from using the TP-PCR forward and reverse primer combinations, respectively. (a) shows an enlarged electropherogram image of sample 58 with repeat lengths 5 and 140; illustrating the ability to visualise the expanded allele peak, within the clinically mild range, at the right-hand end of the characteristic ladder profile. (b) shows two clear allele peaks illustrating the ability to size alleles within the unaffected range with the TP-PCR reverse primer combination.

A complete characterisation of each DNA sample including age, sex, repeat length, disease classification, and methods of analysis in the validation study is shown in Table 2 (rows 1–21). A test study followed using 53 further DNA samples that were amplified using conventional PCR and the forward and reverse TP-PCR primer combinations (see rows 22–74 of Table 2). Conventional PCR amplification allowed the sizing of up to 150 CTG repeats, thereby confirming a clinical diagnosis of mild DM. Taken together, the TP-PCR primer combinations were able to confirm the size of the lower alleles identified in the conventional PCR amplification and detect the presence of (but not accurately size) expanded alleles to distinguish between mild and classic DM. The TP-PCR forward primer combination showed a clear peak at the end of the characteristic ladder profile for smaller expansions within the mild range (Figure 5(a)). Four of the 53 DNA samples showed 3′ interruptions in the TP-PCR forward primer combination electropherograms; however, the TP-PCR reverse primer combination electropherograms showed clear expanded profiles. The TP-PCR reverse primer combination was able to accurately size alleles within the unaffected range consistent with the conventional PCR and SB repeat scores where available (Figure 5(b)).

## 4. Discussion

TP-PCR methods described in the literature use a P3 primer to prevent progressive shortening of the PCR products during subsequent cycles. The data presented above does not show signs of progressive shortening of PCR products. There is adequate amplification across the repeat sequence to enable accurate scoring of alleles within the normal to mild disease range and accurate scoring of the presence and absence of expansions within the classical and congenital ranges of disease without primer P3. Also, the TP-PCRs without P3 showed stronger amplification across the repeat sequence.

The P4CTG primer used in this study was designed against the human *HD* gene repeat locus, with a 5′ sequence that is complementary to that immediately downstream of the *HD* gene CAG repeat [10]. The intention of this specific tail region was to enhance the potential to amplify both the normal allele and the expanded alleles at the *HD* locus. This primer contrasts with those used in other TP-PCR methods described in the literature. These methods use a nonspecific tail sequence to create a unique platform for the binding of primer P3 in order to enable sufficient amplification within a CTG repeat hyper-expansion. Critically, as the trinucleotide repeat in both Huntington disease and myotonic dystrophy is a CTG sequence, a simple change of primer P1 to one that is specific to the upstream of the *HD* gene repeat enables the use of the P4CTG primer for diagnostic testing of repeat expansions in both DM-1 and HD.

Due to the presence of interruptions observed at both the 3′ and the 5′ ends of the CTG repeat sequence in the DM-1 locus, neither the forward nor the reverse primer combination can be considered reliable for clinical diagnostic testing alone, so they should be used in tandem. Although not shown here, it is possible to combine the TP-PCR forward and reverse primer combinations in the same reaction using different labels in order to streamline diagnostic testing further.

On validation of the TP-PCR forward and reverse primer combination, these primers were successfully used in concert with conventional PCR in a diagnostic setting, thereby providing a simple and effective method to accurately size alleles below 150 repeats and clearly indicate the presence of expanded alleles.

In conclusion, the combination of the forward and reverse TP-PCR primer combinations provide a simple and effective means of identifying the presence or absence of expanded CTG repeat alleles in the *DMPK* gene. The same P4CTG primer has been shown to work both with myotonic dystrophy and Huntington disease repeat sequences, streamlining the work flow for these diseases.

## Figures and Tables

**Table 1:** Primer sequences.

Primer name	Primer sequence
P1-FAM	FAM-CTTCCCAGGCCTGCAGTTTGCCCATC
P2	AACGGGGCTCGAAGGGTCCTTGTAGC
P4CTG	GGCGGTGGCGGCTGTTGCTGCTGCTGCTGC
P3	GGCGGTGGCGGCTGTTG
P2-FAM	AACGGGGCTCGAAGGGTCCTTGTAGC
P4CAG	GGCGGTGGCGGCTGTTGCCAGCAGCAGCAGCAG

**Table 2:** Characterisation of samples, including age, sex, repeat length, analysis methods used, and disease classification.

Sample	Age	Sex	Repeat length	Analysis methods used			Disease classification
				PCR	SB	TP-PCR	
1	1 y 7 m	F	7, 11	*√*	*√*	*√*	Unaffected
2	2 y	F	11, 13	*√*	*√*	*√*	Unaffected
3	3 y	M	12, 13	*√*	*√*	*√*	Unaffected
4	5 y	F	5, 14	*√*	*√*	*√*	Unaffected
5	48 y	F	11, 15	*√*	*√*	*√*	Unaffected
6	85 y	M	11, 26	*√*	*√*	*√*	Unaffected
7	11 m	M	5, 27	*√*	*√*	*√*	Unaffected
8	72 y	M	13, 57	*√*	*√*	*√*	Mild
9	68 y	F	5, 74	*√*	*√*	*√*	Mild
10	34 y	F	4, 77	*√*	*√*	*√*	Mild
11	57 y	M	11, 84	*√*	*√*	*√*	Mild
12	47 y	F	5, 120	*√*	*√*	*√*	Mild-classical
13	45 y	M	5, EXP (250)	*√*	*√*	*√*	Classical
14	57 y	F	5, EXP (380) (I)	*√*	*√*	*√*	Classical
15	10 y	M	12, EXP (500)	*√*	*√*	*√*	Classical
16	24 y	M	14, EXP (550)	*√*	*√*	*√*	Classical
17	21 y	F	12, EXP (800)	*√*	*√*	*√*	Classical
18	38 y	F	22, EXP (1000–1500)	*√*	*√*	*√*	Classical
19	36 y	M	5, EXP (1067–1600)	*√*	*√*	*√*	Classical
20	11 y	F	5, EXP (400)	*√*	*√*	*√*	Classical
21	54 y	F	24, EXP (260–490) (I)	*√*	*√*	*√*	Classical
22	2 M	M	13, 13	*√*		*√*	Unaffected
23	1 d	M	5, 13	*√*		*√*	Unaffected
24	24 y	M	5, 11	*√*		*√*	Unaffected
25	27 y	M	5, 10	*√*		*√*	Unaffected
26	6 M	F	5, 5	*√*		*√*	Unaffected
27	54 y	F	14, 15	*√*		*√*	Unaffected
28	52 y	F	23, 27	*√*		*√*	Unaffected
29	25 y	F	5, 12	*√*		*√*	Unaffected
30	23 y	F	11, 13	*√*		*√*	Unaffected
31	32 y	M	5, 5	*√*		*√*	Unaffected
32	23 y	M	12, 14	*√*		*√*	Unaffected
33	56 y	M	8, 12	*√*		*√*	Unaffected
34	16 y	F	12, 20	*√*		*√*	Unaffected
35	21 y	F	11, 13	*√*		*√*	Unaffected
36	32 y	M	13, 14	*√*		*√*	Unaffected
37	3 y	F	5, 5	*√*		*√*	Unaffected
38	1 y 8 m	F	5, 13	*√*		*√*	Unaffected
39	33 y	F	5, 5	*√*	*√*	*√*	Unaffected
40	16 y	F	5, 13	*√*		*√*	Unaffected
41	70 y	F	5, 5	*√*		*√*	Unaffected
42	23 y	F	5, 13	*√*		*√*	Unaffected
43	37 y	M	5, 14	*√*		*√*	Unaffected
44	57 y	M	5, 13	*√*		*√*	Unaffected
45	65 y	F	5, 12	*√*		*√*	Unaffected
46	30 y	F	5, 14	*√*		*√*	Unaffected
47	55 y	M	15, 62	*√*		*√*	Mild
48	61 y	M	14, 89	*√*		*√*	Mild
49	32 y	M	26, 84	*√*		*√*	Mild
50	53 y	F	5, 81	*√*		*√*	Mild
51	63 y	F	15, 50	*√*		*√*	Mild
52	54 y	M	5, 97	*√*		*√*	Mild
53	39 y	F	13, 51	*√*		*√*	Mild
54	73 y	M	13, 56	*√*		*√*	Mild
55	37 y	F	5, 101	*√*		*√*	Mild-classical
56	47 y	M	5, 141	*√*	*√*	*√*	Mild-classical
57	60 y	F	13, 92–145	*√*	*√*	*√*	Mild-classical
58	30 y	M	5, 140	*√*	*√*	*√*	Mild-classical
59	43 y	M	5, EXP	*√*		*√*	Classical
60	6 y	F	5, EXP	*√*		*√*	Classical
61	22 y	M	5, EXP	*√*		*√*	Classical
62	27 y	F	11, EXP	*√*		*√*	Classical
63	30 y	M	5, EXP	*√*		*√*	Classical
64	55 y	M	13, EXP (I)	*√*		*√*	Classical
65	3 y	M	12, EXP	*√*		*√*	Classical
66	80 y	F	10, EXP (I)	*√*	*√*	*√*	Classical
67	42 y	F	5, EXP	*√*		*√*	Classical
68	50 y	M	13, EXP	*√*		*√*	Classical
69	24 y	F	21, EXP	*√*		*√*	Classical
70	42 y	M	5, EXP	*√*		*√*	Classical
71	52 y	F	12, EXP	*√*		*√*	Classical
72	72 y	M	19, EXP (I)	*√*		*√*	Classical
73	29 y	F	5, EXP	*√*		*√*	Classical
74	45 y	F	5, EXP (I)	*√*		*√*	Classical

Repeat lengths up to 150 repeats are shown, and expansions (EXP) are indicated where the specific size of the allele is no longer able to be sized accurately (in excess of 150 repeats). I indicates samples that have a 3′ interruption using the TP-PCR forward primer combination. Ticks indicate which methods were used to analyse each sample. PCR: standard polymerase chain reaction; SB: Southern blot; TP-PCR: triplet repeat primed PCR.
